# Resistance to immune checkpoint therapies by tumour-induced T-cell desertification and exclusion: key mechanisms, prognostication and new therapeutic opportunities

**DOI:** 10.1038/s41416-023-02361-4

**Published:** 2023-07-15

**Authors:** Mona Meng Wang, Sarah E. Coupland, Tero Aittokallio, Carlos R. Figueiredo

**Affiliations:** 1 Medical Immune Oncology Research Group (MIORG), Institute of Biomedicine, Faculty of Medicine, University of Turku, Turku, Finland; 2 Singapore National Eye Centre and Singapore Eye Research Institute, Singapore, Singapore; 3 InFLAMES Research Flagship Center, University of Turku, Turku, Finland; 4 Liverpool Ocular Oncology Research Group (LOORG), Institute of Systems Molecular and Integrative Biology, Department of Molecular and Clinical Cancer Medicine, University of Liverpool, Liverpool, UK; 5 Institute for Molecular Medicine Finland (FIMM), HiLIFE, University of Helsinki, Helsinki, Finland; 6 Institute for Cancer Research, Department of Cancer Genetics, Oslo University Hospital, Oslo, Norway; 7 Oslo Centre for Biostatistics and Epidemiology (OCBE), Faculty of Medicine, University of Oslo, Oslo, Norway; 8 Turku Bioscience Centre, University of Turku, Turku, Finland

**Keywords:** Immunotherapy, Tumour immunology

## Abstract

Immune checkpoint therapies (ICT) can reinvigorate the effector functions of anti-tumour T cells, improving cancer patient outcomes. Anti-tumour T cells are initially formed during their first contact (priming) with tumour antigens by antigen-presenting cells (APCs). Unfortunately, many patients are refractory to ICT because their tumours are considered to be ‘cold’ tumours—i.e., they do not allow the generation of T cells (so-called ‘desert’ tumours) or the infiltration of existing anti-tumour T cells (T-cell-excluded tumours). Desert tumours disturb antigen processing and priming of T cells by targeting APCs with suppressive tumour factors derived from their genetic instabilities. In contrast, T-cell-excluded tumours are characterised by blocking effective anti-tumour T lymphocytes infiltrating cancer masses by obstacles, such as fibrosis and tumour-cell-induced immunosuppression. This review delves into critical mechanisms by which cancer cells induce T-cell ‘desertification’ and ‘exclusion’ in ICT refractory tumours. Filling the gaps in our knowledge regarding these pro-tumoral mechanisms will aid researchers in developing novel class immunotherapies that aim at restoring T-cell generation with more efficient priming by APCs and leukocyte tumour trafficking. Such developments are expected to unleash the clinical benefit of ICT in refractory patients.

## Introduction

The treatment of advanced metastatic solid tumours has achieved a new milestone with the approval of immune checkpoint therapies (ICT) targeting immune checkpoint regulators such as CTLA-4 (cytotoxic T-lymphocyte antigen 4), PD-1 (the programmed cell death-1) and others [1, 2]. Immunogenic tumours (such as metastatic skin melanoma) often respond to ICT. The 5-year survival rates for melanoma reached an unprecedented 52% when applying the combined CTLA-4 and PD-1 blockade approach [3]. However, many patients with advanced solid tumours do not respond well or at all to ICT (refractory patients). Patients responding to ICT and reaching durable responses initially develop normal physiological immune responses directed against their tumours. These patients generate effector cytotoxic T cells (CTLs), followed by the generation of memory T cells (T_EM_), which has been recently associated with durable responses following tumour-antigen re-exposure [4]. Mechanistically, ICT works by enhancing the anti-tumour effector functions of CD4+ and CD8 + T cells [5], initially formed during their first contact (*priming*) with tumour peptides complexed with major histocompatibility complex (MHC) molecules.

**Fig. 1:**
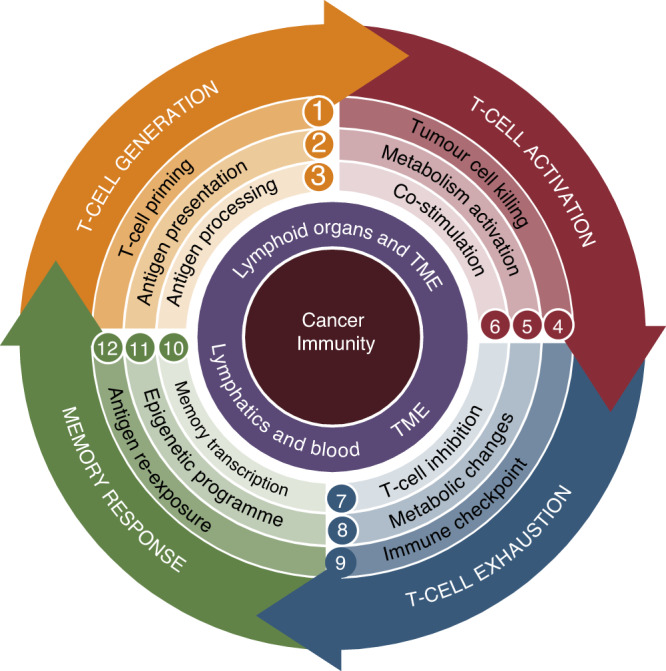
Wheel diagram of cancer immunity. Wheel fragments/colours represent critical immune stages that enable the wheel’s movement towards T-cell exhaustion for ICT benefit and memory for durable responses.

Therefore, the first conditional rule for ICT response is the successful MHC-dependent presentation of tumour antigen in the format of peptides to T-cell receptors (TCR), which results in the generation of a clonally expanded population of anti-tumour effector lymphocytes, as shown in the wheel diagram of Fig. 1 (orange, Stages 1–3). The diagram provides an overview of the complete cancer immunity process. From the T-cell generation stage, it emphasises crucial events that are essential for achieving clinical benefits from ICT, such as the activation of T cells (red, Stages 4–6), T-cell exhaustion (blue, Stages 7–9), and memory response (green, Stages 10–12), also reviewed in [6]. In brief, efforts attempting to improve ICT response in relapsed patients in clinical trials are currently centred on improving T-cell effector functions, targeting new immune checkpoint regulators (e.g., lymphocyte-activation gene 3 (LAG-3)) and co-stimulatory agonists (e.g., inducible T-cell co-stimulator (ICOS) agonism), as well as boosting the memory phenotype using epigenetic modulators.

In addition to these innovative approaches, there is a growing interest in dissecting resistance mechanisms that impair the generation of anti-tumour T cells for the rational development of new combinatorial approaches, with the aim of unleashing cancer immunosurveillance to refractory tumours (Fig. 1, Stages 1–3). Poorly immunogenic tumours are not necessarily those with low tumour mutational burden (TMB) since low TMB does not necessarily exclude immunogenic responses nor ensure the response to ICT [6]. Cancer immunogenicity primarily depends on two factors: (i) the existence of tumour antigens as products of tumour genetic instabilities (mutations); and (ii) a functional antigen processing and priming machinery in both antigen-presenting cells (APCs) and tumour cells [7].

**Fig. 2:**
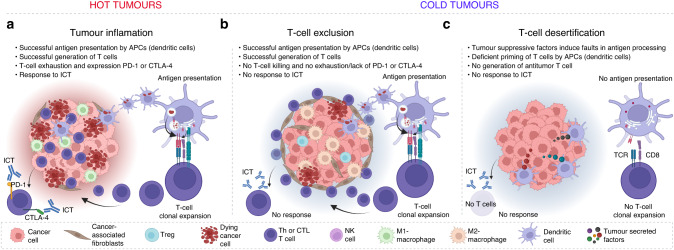
Cold and hot tumours. General aspects of innate and adaptive immunity for ICT outcome. **a** Inflamed tumours: cancer cells often lack strategic mechanisms inducing immunosuppression that leads to T-cell desertification and exclusion, and consequently, these tumours respond well to ICT. **b** Desert tumours: cancer cells have poor mutational levels. In addition, they can trigger immunosuppressive mechanisms that impair the efficient priming of T cells by tumour cells and APCs. These tumours are universally refractory to ICT. **c** T-cell-excluded tumours: cancer cells do not efficiently suppress antigen processing and presentation, and these tumours can elicit immunogenic responses. However, cancer cells can influence local TAMs and CAFs to block the infiltration of anti-tumour T cells. This resistance mechanism to ICT can be innate in refractory tumours or acquired in relapsed tumours.

Indeed, it has been shown that the ICT response correlates with dense tumour infiltration of T lymphocytes in advanced solid tumours, characterised mainly by high intratumor levels of CD4 and CD8 positive markers in histological sections [8]. These tumours are frequently termed ‘hot’ because they exhibit an inflamed characteristic with infiltrated immune cells (Fig. 2a). In contrast, non-inflamed tumours are universally refractory to ICT and are therefore classified as ‘cold’ (Fig. 2b, c). Cold tumours can be further divided into two subgroups: ‘desert’ tumours, which are deficient in generating anti-tumour T cells and have very few numbers of infiltrated T cells in the tumour stroma [9] (Fig. 2b), and ‘excluded’ tumours, where existing anti-tumour T cells are excluded from the tumour stroma and remain trapped in the tumour periphery [10] (Fig. 2c).

In this review, we delve deeper into the concept that the effectiveness of T-cell priming by DCs in cancer immunosurveillance is of paramount importance, rather than solely relying on high TMB, for enhancing tumour immunogenicity and initial responsiveness to ICT. We will specifically highlight the therapeutic hurdles faced in revitalising DCs in desert tumours and offer insights into the current advancements, both in pre-clinical and clinical stages, for novel ICT combination strategies. This perspective forms the basis of our ensuing discussion.

## Desert tumours and resistance to ICT

TMB has emerged as a potential underlying cause of ‘cold’ tumours and resistance to the ICT [11]. TMB refers to the number of mutations present in the DNA of tumour cells, and higher TMB has been associated with increased immunogenicity and the generation of neoantigens for immunotherapy efficacy, reviewed in ref. [12]. However, paradoxically, tumours with high TMB may still exhibit a lack of response to ICT, indicating the presence of additional immunosuppressive mechanisms. Understanding the intricate relationship between TMB and the immune microenvironment is crucial for unravelling the factors contributing to immune evasion and resistance to therapy in these tumours. Further research is needed to elucidate the complex interplay between TMB, processing of tumour antigens APCs for further immune cell infiltration, and the immunosuppressive mechanisms present within the tumour microenvironment (TME), for the rational development of innovative therapeutic strategies to overcome resistance and enhance the efficacy of immunotherapies.

Cancer immunoediting postulates that immunologically sculpted tumours may induce specific modulation of immune cell functions, including suppression of DCs maturation and activation, which in turn, provides the tumours with an improved ability to prevent the generation of CTLs and escape immunosurveillance [7]. Desert tumours can hide from the immune system and become universally refractory to ICT (Fig. 2b). Impaired antigen processing and presentation by tumour cells are known at the level of downregulation of MHC class I (HLA-A in humans) in response to an immune-suppressed TME. This is known to impact ICT outcomes, such as loss of beta-2-microglobulin (β2M) expression that affect the cell surface expression of HLA-A, which in turn impairs antigen presentation to CTLs [13]. Faults in HLA class I antigen processing machinery and how this impact anti-tumour immunity promoted by existing CTLs have been reviewed [14]. In this current review, we focus on the potential causes of tumour desertification, considering faults in antigen processing and presentation by MHC class II machinery in APCs (mainly DCs), which result in deficient T-cell priming and generation of CTLs for ICT benefit. The antigen presentation machinery can be targeted through several tumour-derived factors and mechanisms, as summarised in Table 1.

**Table 1:** Summary of central mechanisms of T-cell desertification and exclusion driving resistance to ICT.

Process	Mechanisms	Impacted ICT	Reference
T-cell desertification	β2M downregulation CD1d/*SPI1* epigenetic control	Anti-PD-1/PD-L1	[56, 58].
	Msr1/Clever-1 upregulation	Anti‐CTLA-4, anti-PD-1/PD-L1	[29, 31]
	*CIITA* downregulation	Anti-PD-1/PD-L1	[15]
	HLA-DM deficiency	Potential resistance to ICT	[16]
	Tumour-derived suppressive cytokines	Anti‐CTLA-4, anti-PD-1/PD-L1	[27, 32, 35–38]
	TGF-β upregulation	Anti-PD-1/PD-L1	[37, 38]
	IDO1 upregulation	Anti-PD-1/PD-L1	[39, 40]
	Tumour-induced STAT3 overexpression	Anti-PD-1/PD-L1	[40]
	WNT/β-catenin upregulation	Anti‐CTLA-4, anti-PD-1/PD-L1	[42, 43, 46, 47, 61]
	COX-2 upregulation	Anti‐CTLA-4, anti-PD-1/PD-L1	[44]
	MIF/CD74 and /CXC axis activation	Anti‐CTLA-4, anti-PD-1/PD-L1	[27, 61, 120]
	*BAP1* deficiency	Anti‐CTLA-4, anti-PD-1/PD-L1	[61]
T-cell exclusion	WNT/β-catenin upregulation	Anti‐CTLA-4, anti-PD-1/PD-L1	[42, 43, 46, 47]
	MHC molecules downregulation	Anti‐CTLA-4, anti-PD-1/PD-L1	[51–53, 55]
	β2M downregulation	Anti‐CTLA-4, anti-PD-1/PD-L1	[56, 57]
	CAFs upregulation	Anti-PD-1, anti-PD-1/PD-L1	[10, 19, 59]

Deficiencies in the antigen presentation machinery of APCs induced by tumour cells are not well known by the scientific community. Researchers have reported deficiencies in the context of regulation of MHC class II gene expression in APCs impacting antigen presentation by downregulation of APC-specific regulator of transcription *CIITA* in large B-cell lymphomas [15]. Similarly, deficiencies in the context of human leukocyte antigen DM (HLA-DM) may also play an essential role in suppressing immunogenicity against a subset of Hodgkin’s tumour cells [16]. HLA-DM is essential in replacing the Class II-associated invariant chain peptide (CLIP) from the MHC class II molecule with tumour peptides that can be presented to T cells [17].

The implications of deficits in CIITA and HLA-DM within APCs are far-reaching and present a roadblock to effective immune response across many cancer types. With *CIITA* expression and HLA-DM functionality compromised, the transcriptional regulation of MHC class II genes and loading of tumour peptides is hindered, which negatively impacts the efficient display of tumour antigens to T cells. Both circumstances culminate in a weakened generation and expansion of anti-tumour T cells, providing a pathway for immune evasion and contributing to the emergence of ‘cold’ tumours or tumour desertification. It is of utmost importance that future investigations cast a wide net, encompassing diverse cancer types, to ascertain the deficit prevalence of *CIITA* expression and HLA-DM functions. Current publicly available RNA and protein datasets from cancer patients treated with different ICT in tandem with cutting-edge genomic and bioinformatic tools could hold provide important information on these deficits and uncover new tumour desertification biomarkers, potentially driving advancements in precision medicine, facilitating the rational design of new therapeutic strategies aimed at restoring CIITA and HLA-DM associated networks, boosting tumour immunogenicity.

Other mechanisms that can potentially impact MHC class II machinery were recently evidenced in tumour-derived signalling that impacts biochemical processes affecting antigen processing and priming of T cells. As an essential cancer immunotherapy research model, metastatic cutaneous melanoma is believed to have sufficient TMB for antigen presentation, immunogenicity, and ICT clinical benefit [11]. Yet, at least 50% of patients do not respond to ICT [18], primarily due to T-cell exclusion and desertification mechanisms [19]. In the context of the pre-clinical experimental B16 cutaneous melanoma model, and despite its significant TMB, this tumour is still considered poorly immunogenic, which may resemble deficiencies in antigen presentation driven by the genetic instabilities of these tumours.

Seventeen years since the first use of granulocyte-macrophage colony-stimulating factor (GM-CSF) tumour vaccine to unleash T-cell generation for anti-CTLA-4 blockade response in B16 melanomas [20, 21], extensive pre-clinical research has been carried out to improve DCs immunogenic functions. When Prof. James P. Allison and his group initially introduced the anti-CTLA-4 blockade to reinvigorate anti-tumour T cells to fight solid tumours, the therapeutic effect of this breakthrough was only achieved in the context of tumours with preserved immunogenicity (hot tumours) [22, 23]. In other words, tumour antigens could be processed and presented by APCs cells for optimal generation of anti-tumour CTLs. Responsive tumour models used in this research, such as fibrosarcoma Sa1/N, 51BLim10, and RENCA, are relatively immunogenic and could be eradicated following the CTLA-4 blockade therapy. These tumour models probably do not have suppressing mechanisms associated with antigen processing and priming of T cells by dendritic cells (DCs). The same was not observed in the metastatic cutaneous B16 melanoma model, despite a significant TMB [21].

Indeed, during the first studies of tumour immunology performed by the fathers of modern tumour immunology [24], it was shown that metastatic melanoma cells could express specific antigens capable of eliciting anti-tumour immune responses [25]. However, these immune responses are not sufficient to eradicate the tumours. Eradication of poorly immunogenic B16 tumours following anti-CTLA-4 blockade was only possible in a synergistic approach using a GM-CSF tumour vaccination [21]. GM-CSF is a critical factor for DC growth, survival, and functional activation. For that reason, poorly immunogenic tumours (desert tumours) may not only be by-products of low TMB, but somehow, they can subvert T-cell generation by suppressing immunogenic functions of DCs when it comes to processing and presenting existing tumour antigens.

One recent research line has led to the discovery of the anti-tumour immunoglobulin complementarity-determining regions (CDRs) derived peptide that could not only suppress the growth of B16 tumours in vivo but also restore the immunogenic capacity of DCs to activate anti-tumour immune responses by CTLs [26]. While studying the immune modulatory mechanism of this therapeutic peptide, researchers found a critical immunoediting mechanism that metastatic B16 tumours use to suppress DCs in the TME. They discovered that metastatic B16 cells secrete high levels of the macrophage inhibitory factor (MIF) in the TME, which in turn suppresses local and systemic DCs functions through the kinase activation of the invariant chain II (CD74), usually expressed on the surface of DCs [27].

When active, this axis can be considered a myeloid (or innate) immune checkpoint regulator since it blocks the generation of adaptive T-cell immune responses by inducing tolerogenic signals in DCs, disturbing the priming and clonal expansion of anti-tumour CD8 + T cells. Therefore, the B16 tumour model and other cold tumours with similar T-cell desertification issues are not likely to naturally respond to ICT and would benefit from combinatorial approaches that block the MIF/CD74 axis to unleash DCs-dependent priming of anti-tumour CD8 + T cells. Similar effects were also observed in the context of glioblastoma and have been further reviewed [28]. The potential of targeting the MIF in melanoma immunotherapy is indeed a promising avenue for exploration. MIF’s role in subverting the immunogenic functions of DCs in melanoma highlights one of the potential key pathways melanoma cells might employ to evade immune surveillance, which has previously been overshadowed due to the focus on boosting GM-CSF levels artificially.

Besides MIF, other upregulated immunosuppressive factors in the TME, such as scavenger receptors, have been described to impact cancer immunogenicity leading to cold tumours. The macrophage scavenger receptor (Msr1) can disrupt DCs’ uptake of extracellular lipids, affecting the presentation of soluble peptides via MHC class II [29], and Clever-1/STAB1, also known as FEEL-1, is a scavenger receptor expressed on immunosuppressive monocytes/macrophages that correlates with improved patient outcomes receiving ICT [30, 31].

Tumour-derived cytokines, including IL-6, IL-10, and transforming growth factor (TGF)-β1, also hinder tumour antigen processing and presentation by DCs. Elevated IL-6 levels downregulate MHC class II expression and IL-12 production, impeding the priming and activation of antigen-specific CD4 + T cells in co-cultures with monocyte-derived DCs [32]. It also directly inhibits CD8 + T-cell function by enhancing N-Glycan Branching to decrease antigen sensitivity [33]. IL-6 has also been reported to play a significant role in immune regulation by independently downregulating Fas and FasL, thereby promoting T-cell survival and maintaining anti-apoptotic factors. These effects could potentially strengthen the anti-tumour immune response. Moreover, early IL-6 signalling can stimulate expansion and cytokine production in primed or memory CD4 + T cells, which could amplify the body’s immune response against infections or diseases [34]. Therefore, while IL-6 has pivotal roles in immune response, inflammation, and survival of T cells, it also has potential drawbacks, including ambiguous effects on T-cell proliferation, possible hindrance of the immune response against tumours, and association with chronic inflammatory conditions and autoimmune diseases. More studies are necessary to fully understand the complex roles of IL-6 in health and disease, which could potentially guide the development of therapeutics that harness its beneficial effects while mitigating the negative ones.

The role of IL-10 in suppressing anti-tumour immune responses is more established. IL-10 disrupts MHC class II assembly, reduces cathepsin S expression, and impairs antigen-MHC class II complex formation in an IFN-γ-dependent pro-inflammatory TME [35]. Furthermore, IL-10 inhibits endo/lysosomal trafficking of antigen-MHC class II complexes, and TGF-β leads to reduced antigen uptake by DCs, both impairing T-cell priming [29, 32, 35–38]. Future directions in cancer immunotherapy should focus on counteracting IL-10’s immunosuppressive effects. Therapeutic strategies could inhibit IL-10 activity to enhance antigen presentation and T-cell activation. This could be achieved by targeting IL-10’s disruption of MHC class II assembly, antigen-MHC class II complex formation, and endo/lysosomal trafficking. Amplifying the IFN-γ-dependent pro-inflammatory TME might also negate IL-10’s suppressive effects. Therefore, a targeted approach against IL-10 with existing gene-therapy tools could provide a more effective cancer immunomodulatory strategy.

Other innate checkpoint regulators of adaptive immune responses that induce T-cell anergy and desertification include expression of indoleamine 2,3-dioxygenase 1 (IDO1) by DCs when exposed to the TGF-β, vascular endothelial growth factor (VEGF), and macrophage colony-stimulating factor (M-CSF) [39]. IDO1 negatively impacts the immunological synapsis of T-cell priming with conventional DCs. In addition to IDO1, it has been long known that tumours can suppress immunogenic signals by activating the signal transducer and activator of transcription 3 (STAT3) signalling, which ultimately suppresses DCs maturation. In ICT resistance, it is believed that hyperactivated STAT3, both in cancer cells and stromal cells, may lead to ICT resistance through various mechanisms of cancer immunity suppression, as reviewed earlier [40].

Through intensive data mining of The Cancer Genome Atlas [41] pan-cancer database for the discovery of biomarkers of resistance associated with T-cell desertification, researchers have initially found that the WNT/β-catenin pathway has importance in this process [42], primarily associated with tumour exclusion of CD103+ DCs, and consequently, antigen-specific T cells [43]. This study showed that specific cancer types, such as renal-, adrenocortical- and ovarian carcinomas, as well as sarcomas, had the highest β-catenin expression enrichment driving T-cell desertification when compared with inflamed tumours. Significantly, β-catenin-dependent modulation has also been described to be impacted by MIF, essentially by inducing tumour expression of cyclooxygenase-2 (COX-2), which reduces intratumor trafficking of CD103+ DCs [44].

Taken together, these findings provide critical updates to our knowledge of how cancer cells use several molecular and cellular mechanisms to suppress local and systemic antigen processing and priming of effector anti-tumour T cells. Desert tumours have stimulated a growing interest in the rational development of combinatorial approaches aimed at co-targeting these mechanisms to unleash anti-tumour T-cell generation and infiltration in desert tumours, discussed further. In addition, it is expected that the development of new prognostication tools that accurately identify patients with desert tumours will optimally guide patient selection to ICT and may further select eligible patients for a new generation of rational combinatorial approaches.

## T-cell exclusion and resistance to ICT

The absence of tumour T-cell infiltration (T-cell exclusion) is one of the leading causes of resistance to ICT in cold tumours [45] (Fig. 2c). The intrinsic tumour biomarkers, such as WNT/β-catenin activation and phosphatase and tensin homologue (PTEN) loss, have been primarily associated with tumour exclusion of CD8 + T cells [42, 43, 46, 47]. Drug development targeting these pathways has been considered for clinical trials to restore the infiltration of excluded tumour-specific T cells [48–50]. One major factor contributing to the exclusion aspect of T cells is the immunosuppressive nature of the TME, especially in the tumour-induced downregulation of MHC molecules. MHC class I and class II proteins play critical roles in efficient antigen expression and are critical for T-cell activation following the priming of tumour antigens [51]. Downregulation of MHC molecules is a clever tactic that tumours find to restrain local anti-tumour immune responses, leading to T-cell anergy and tolerogenesis [52].

Indeed, loss of MHC class I levels has been associated with poor response to anti-CTLA-4 blockade therapy using ipilimumab. Still, it does not predict resistance to the anti-PD-1 blockade therapy using nivolumab or the combination using ipilimumab with nivolumab [53]. On the other hand, increased expression of MHC class II in the TME is associated with improved response to nivolumab but not with ipilimumab alone or in combination with nivolumab. These findings initially suggested that MHC downregulation specifically impacts the outcome of different ICT [54]. This was further confirmed in pre-clinical studies, where distinct cellular and molecular mechanisms were associated with anti-PD-1 and anti-CTLA-4 monotherapies [55].

Resistance to ICT has also been attributed to tumour downregulation of β2M, an essential component of MHC class I antigen presentation, suggesting that deficiency in antigen presentation to CD8 + T cells is another form of ICT resistance [56]. However, the loss of antigen presentation in this context should not be mixed with a poor generation of anti-tumour T cells by APCs in desert tumours. β2 M downregulation, as a resistance mechanism to ICT, is restricted to tumour cells that have reduced tumour-antigen presentation via MHC class I. Therefore, existing clonally expanded anti-tumour CD8 + T cells, which are eventually able to reach the tumour site, will have a reduced tumour-antigen interaction via MHC class I and TCR, in addition to co-stimulatory molecules, reaching poor activation signals, which ultimately results in drastic T-cell proliferation suppression [56]. This can be explained by the fact that optimal T-cell exposure to tumour antigens via MHC class I or II results in more potent and prolonged TCR activation signals, whereas lower antigen exposure results in shorter interactions, transmitting weaker and insufficient TCR signals [57]. Therefore, the latter cells are excluded from tumours predominantly by tumour-induced immunosuppressive mechanisms.

However, a role for β2M downregulation as a consequence of desert tumour formation in ICT resistance has been recently uncovered. Researchers have shown that deficient β2M expression impairs DCs from effectively presenting glycolipid antigens through CD1d to Natural Killer T cells (NKTs), thereby affecting NKT levels in the TME [58]. In addition, researchers have identified epigenetic changes that affect not only β2M expression but also *SPI1*, a crucial regulator of CD1d expression. This research aligns with the growing understanding of epigenetic alterations as a factor contributing to ICT resistance [6], providing a rationale for developing novel combination treatments targeting epigenetic regulators of CD1d/*SPI1* in metastatic melanoma.

In addition to immunosuppression, T-cell exclusion is characterised by observing anti-tumour T cells trapped in the tumour margin, where CAFs display an essential role [59]. Indeed, tumours that induce T-cell exclusion mechanisms often impair infiltration of T cells by creating a fibrotic shield due to the interplay of secreted factors from cancer-associated fibroblasts (CAFs), which have a myofibroblastic phenotype and tumour-associated macrophages (TAMs) [10]. Anti-CTLA-4 treatment has been reported to improve the infiltration of existing T cells in tumours with T-cell exclusion resistance mechanisms, thus improving responses to anti-PD-1 treatment [6]. Spatial immune profiling of liver metastasis of metastatic uveal melanoma (mUM), one of the most refractory cancer types to ICT, revealed that IDO1 and β-catenin overexpression might also play an important role in trapping tumour-infiltrating lymphocytes [60] within peritumoral fibrotic areas in the liver [61]. This has been further investigated in other cancer types that develop metastasis in the liver and are poor responders to ICT, such as metastatic pancreatic ductal adenocarcinoma (PDAC) [62].

During liver fibrosis formation, cancer-associated fibroblasts (CAFs) acquire a myofibroblast phenotype expressing α-smooth muscle actin (αSMA), usually associated with collagen and fibrosis. In cancer lesions with high levels of CAFs with a “fibrosis-inducing” phenotype, anti-tumour CD8 T cells fail to infiltrate and accumulate at the tumour margin, resulting in resistance to multiple immunotherapies, including ICT [10]. In addition, TAMs with an alternative alternatively activated macrophage (M2)-like phenotype have been extensively reported to mediate fibrosis, primarily by exerting a reciprocal interaction with myofibroblasts. Mechanistically, stellate tissue cells attract and stimulate macrophages with cytokines, including the M-CSF. Transformed macrophages will influence local fibroblasts by secreting fibrosis-inducing factors, including TGF-β1 and platelet-derived growth factor (PDGF) [19, 63]. The physical exclusion of CD8 + T cells from the tumour site has been demonstrated to be a critical limiting factor for ICT approaches [64].

The study of T-cell exclusion and resistance to ICT has revealed important insights, but much remains unknown about the underlying mechanisms of T cells. While intrinsic tumour biomarkers and the TME have driven T-cell exclusion insights, there is still a limited understanding of the complex factors involved. Current multi-omic approaches and technologies enable the evaluation of retrospective tumour samples that hold answers for tolerogenic mechanisms of innate immunity, with emerging data mining AI tools, however, hold the potential to unlock new insights and provide a deeper understanding of T-cell exclusion mechanisms. Future directions should explore the rational discovery of novel biomarkers using a functional approach that minimises noise and maximises prediction consistency across different cancer types.

## Prognostic biomarkers of T-cell desertification for ICT outcome

More than half of cancer patients treated with ICT, including initially refractory patients, develop resistance to ICT, highlighting the urgent need to identify predictive biomarkers of short- and long-term clinical efficacy. However, a significant challenge in the field of ICT is the scarcity of predictive biomarkers that are robust in prediction accuracy and consistent across different cancer cohorts, meaning that they should also predict responses across various cohorts other than those where they are exhaustively trained and initially validated. This may require different sets of biomarkers and second-line therapies, such as combination treatments, to address the diverse patient population.

Predictive biomarkers for ICT response have been initially proposed as tumour-intrinsic factors, or as phenotyping immunohistochemistry biomarkers, such as programmed death-ligand 1 (PD-L1) expression on tumour and immune cells [65] and CD8+ TILs [66]. However, the prediction of ICT outcomes based on PD-L1 expression has shown to be limited, due to our limited understanding of its biological context and expression site, in addition to technical limitations. Although the presence of CD8+ TILs has been associated with good ICT outcomes [67], methods for using its predictive power across different cancer types lack clinical validation. In addition, some studies suggest that the location of T cells within the tumour and their activation status needs proper investigation [68, 69]. For example, high TILs levels in biopsies of primary uveal melanoma (UM) tumours have been associated with poor outcomes [70]. The differentiation and activation levels of CD8 + T cells in this ICT refractory cancer type revealed a regulatory phenotype (HLA-DR + CD38 + CTLA-4- CD8 + T cells) rather than a cytolytic one [61], suggesting that the immune-privileged aspect of some organs, such as the eye and liver, can suppress T-cell activation and exhaustion, which make them unresponsive to ICT. Therefore, the more we fill gaps in our current understanding of the anti-tumour immunity process, the more we acknowledge that single biomarkers in isolation are insufficient to predict ICT outcomes.

Integrating multi-omics data from tumours linked with available clinical data collected from multicentric trials enables the development of machine learning-based biomarkers for the prognostication of the ICT outcome [71, 72]. Biomarkers revealed through these signatures can partly contribute to understanding the anti-tumour immunity process. For example, specific M2 macrophage transcriptomic signatures associated with stabilin-1 (*STAB1*) have been described to predict resistance to ICT in cold tumours of the Pancreatic Cancer Action Network (PanCAN)-GDC-TCGA study in a T-cell dysfunction manner, meaning that T-cell dysfunction, rather than T-cell trafficking, could be triggered by specific macrophage gene expression programme that impacts T cells functions and their levels within ICT-treated tumours [31]. In ovarian cancer, an 18-gene signature (including *TAP1, ICOS, CD2 COL5A2*) has been described to immunologically characterise desert tumours in coherence with histopathological analysis for intratumor T-cell levels [73]. An immunotherapy signature of 166 immune genes, including IFNγ-inducible genes such as *IDO1, JAK/STAT*, and *HLAs*, as well as checkpoint genes such as *LAG3, CTLA-4, ICOS* and *PD-L1*, was increased in patients with T-cell inflamed tumours, as opposed to desert tumours, suggesting that performing a gene expression profiling and PD-L1 correlation screen before patient’s entry to ICT could predict the patient’s likelihood to respond to ICT in clinical trials [74]. Importantly, *SOCS1* gene was previously identified to regulate *JAK/STAT* and suppress melanoma immunogenicity, and its pharmacological inhibition is now considered a potential target for immunotherapies [75]. Finally, a transcriptional DCs programming signature for enhanced MHC class II antigen presentation has been detected [76], and can be further expanded for developing innovative correlated transcriptomic signatures that optimally predict antigen presentation associated with priming of helper T cell, CTL responses, and ICT outcomes. Altogether, these biomarkers hold the promise of commonality across different cancer types as reference maps orienting the development of new multi-omic machine learning-based tools. However, clinical validation is crucial, considering performance across diverse cohorts, correlation with histopathological analysis, and validation against known immunogenicity-related genes.

## Prognostic biomarkers of T-cell exclusion for ICT outcome

Predicting ICT resistance by T-cell exclusion is a significant challenge. The bulk transcriptomic data from cutaneous melanoma cells have been initially used to identify a transcriptomic programme associated with T-cell exclusion and immune evasion following ICT resistance [77]. Researchers found upregulated and downregulated transcriptomic signatures driving T-cell exclusion that impacts different molecular processes, such as modulation of cyclin-dependent kinase 4 and 6 (CDK4/6) functions [78]. Notably, transcriptomic signatures of immune cells, such as macrophages, were not associated with the ICT resistance by T-cell exclusion but were linked with the response of macrophages to T-cell abundance rather than a cause of T-cell exclusion [77]. Statistical modelling of factors that exclude T-cell infiltration into ICT-treated tumours was also carried out using expression signatures from immunosuppressive cells. The resulting tumour-immune dysfunction and exclusion (TIDE) score is one of the first multigene signatures that predicts the outcome of cancer patients treated with first-line anti-PD-1 or anti-CTLA-4, instead of using single biomarkers like PD-L1 or tumour mutation load [79]. However, this statistical model is exclusively built to predict the outcome of a cohort of patients, which is helpful for clinical trial designs but not applicable to an individualised prognostication approach.

Finally, the cancer research community needs to tackle the challenges associated with biomarker discovery for cold tumour prognostication. Among these challenges is the need for prognostication models that demonstrate reliable prediction performance across diverse randomised subsets of the same cancer type, extending beyond the cohorts initially utilised for model training and development [80]. In addition, the ability to recover rare immune infiltrated cells in desert tumours, such as those that develop in immune-privileged tissues (e.g., eye, testis and brain), poses unique challenges for most microfluidics-based immune profiling technologies since multiple cancer cell types might have a promiscuous expression of some immune genes [81]. New microfluidic techniques have been developed to detect sensitive molecular changes in response to ICT [82]. For example, researchers have developed the single-cell DEPArray platform that performs automated isolation of rare immune infiltrated cells from intraocular and liver biopsies of primary and metastatic UM, respectively, [83, 84]. This platform is expected to address the clinical needs for single-cell based precision immune prognostication in the clinical setting in the near future.

## New mechanisms of T-cell desertification and exclusion: lessons from metastatic uveal melanoma

In striking contrast to cutaneous melanoma, one of the most responsive cancer types to ICT, mUM is universally refractory to ICT due to both T-cell desertification and exclusion issues [11], despite uveal melanocytes being derived embryologically from similar cells to their counterparts in the skin. For example, nivolumab treatment in patients with advanced, treatment-refractory skin melanoma yielded favourable overall survival (OS) rates comparable to those reported in similar patient populations in the literature. Durable responses were observed, even after drug discontinuation, and the long-term safety profile was acceptable [85]. Such response rates have not been seen in mUM [86]. Consequently, mUM is an ideal model for discovering new molecular pitfalls associated with ICT resistance in cold tumours. Despite low mutational loads, as a suggestion of the T-cell desertification process, the presence of tumour-infiltrating lymphocytes (TILs) and clinical responses to adoptive TIL transfer [87], suggests the existence of UM’s immunogenicity is impacted by mechanisms of local immune suppression.

BRCA1-associated protein 1 (*BAP1*) deficiency, primarily caused by its expression losses and mutations, triggers molecular mechanisms that drive tumour growth and impact UM outcome, making it a powerful prognostic biomarker [88–93]. We demonstrated that *BAP1* loss in UM correlated with the upregulation of immune suppressive genes, building an immune suppressive axis, including HLA-DR, CD38 and CD74. Our study was the first immune landscape study of human primary and metastatic UM using state-of-the-art approaches, such as spatial NanoString GeoMX and high-dimensional single-cell mass cytometry (CyTOF) [61]. It revealed that primary and metastatic UM share a similar immune landscape in the context of differential gene expression of *BAP1* [61]. Mass cytometry analysis of pUM confirmed that tumour-infiltrated CD8 + T lymphocytes acquire a regulatory phenotype, as monocytes also turn into tumour-associated macrophages (TAMs). Similar to cutaneous melanoma, the MIF/CD74 axis is also engaged in UM liver metastasis with a deficiency in BAP1 expression.

Furthermore, β‐catenin upregulation appears upregulated in mUM, suggesting tumour activation of molecular pathways associated with DCs deficiencies, immune exclusion, and ICT resistance in mUM. Finally, our study showed a fibrosis-dependent T-cell exclusion mechanism in which TAMs and TILs were entrapped in peritumoral fibrotic areas expressing IDO1, PD-L1, and β-catenin (CTNNB1), suggesting tumour-driven immune exclusion, which can be associated as one of the causes of resistance to ICT in mUM with *BAP1* losses. Dissecting the molecular mechanisms of immune suppression that are governed by the *BAP1* losses can offer a potential roadmap for the development of new combinatorial immunotherapeutic strategies for mUM.

Further research by this team showed an expanded insight into the mechanisms impacting immune transformation in high-risk mUM due to BAP1 loss [94]. It investigated the role of adipophilin, a structural protein in fat storage, in UM [94, 95]. We found that loss of adipophilin expression was associated with poorer survival outcomes in patients with UM, especially in those patients with UM demonstrating loss of nuclear BAP1 expression. A metabolic shift in the TME of mUM associated with adipophilin losses dependent on BAP1 was demonstrated. Using a multidisciplinary approach, we further identified small molecules with potential therapeutic applications, including adrenergic, retinoid, and glucocorticoid receptor agonists, MEK, and RAF inhibitors that effectively reversed the expression of the metabolic multigene signature in UM cells, with carvedilol restoring adipophilin levels. These discoveries highlight the importance of understanding the dysregulated metabolic processes in UM that impact the immune response and suggest that targeting these pathways could represent a promising strategy for improving the response to immunotherapy in this challenging disease.

In concordance with these results, another research team discovered that beta-blockers, already recognised as the gold standard for treating other types of tumours like infantile hemangiomas, have not yet been examined for their effectiveness in treating UM [96]. In this study, researchers also found that carvedilol was effective in blocking tumour-cell viability and the survival of UM cells, which has additive effects on other therapeutic strategies. Altogether, these findings show for the first time a collection of potential repurposed drugs that can reverse the metabolomics of mUM, aiming to unleash the benefit of anti-tumour immune responses and maximise the outcome of ICT in future trials for mUM.

The management of UM patients has seen significant advancements in recent years, particularly in developing new therapeutic approaches such as tebentafusp [97–99]. This ground-breaking bispecific treatment strategy has shown promising results in recapitulating hot tumour features in UM. Moreover, discoveries oriented by BAP1 biology are expected to continue to improve the diagnosis, prognosis, and management of mUM patients in the future, providing new hope for patients with this devastating disease.

Some of these discoveries may be based on experiments using pre-clinical models of UM. These include the chick embryo chorioallantoic membrane (CAM) model, drosophila, zebrafish, mice, rats, rabbits, and others [100–104]. Not only can these pre-clinical models be used to screen drugs (either singularly or in combination), but they can also be used to determine how the host responds to tumours immunologically or to examine how certain factors contribute to tumour growth. A significant advance in UM pre-clinical models has been using patient-derived UM xenografts, which consider tumour heterogeneity, including the mixed cell composition of both primary and metastatic UM [103]. 3D co-cultured UM spheroids also allow replicating the interactions of UM cells with inflammatory cells as well as hepatocyte stellate cells and liver parenchyma and for rapid drug testing [92]. Each model has its limitations, and each can only address ‘part of the question’, so the resulting data must be interpreted within the framework of the limitations of the assay used [104]. However, these methodologies and technologies promise hope of treatment breakthroughs for UM patients with disseminated disease.

## Rational combinations to overcome T-cell desertification and exclusion in cancer

Therapeutic approaches toward unleashing immunogenic responses in cold tumours remain a significant challenge for ICT. Faults in antigen processing and T-cell priming by APCs leading to deficient generation of anti-tumour T cells and, consequently, tumour-immune desertification process are still poorly understood. Attempts to optimise antigen presentation machinery in APCs and T-cell priming are primarily centred on DCs vaccinations, ex vivo approaches, and combinatorial treatments blocking tumour immunosuppressive factors [48, 105].

Efforts to improve DC vaccination include enhancing DC maturation, antigen loading, antigen selection, and presentation [106, 107]. Of note, pre-clinical evidence shows that agonism of stimulator of interferon response cyclic guanosine monophosphate–adenosine monophosphate (cGAMP) interactor 1 (STING) or Toll-like receptors (TLR) enhance the levels and functions of CD103+ DCs, which are the ones responsible for transporting intact antigens to the lymph nodes and priming tumour-specific CD8 + T cells [108–110]. Personalised neoantigen-loaded monocyte-derived dendritic cell (Neo-MoDC) vaccines followed by combination therapy with ICT have shown to be a promising approach. The Neo-MoDC vaccine triggered T-cell responses against neoantigens, and the following combination therapy led to complete regression of all tumours for over 25 months. This suggests that Neo-MoDC vaccines, in combination with ICT, could be a promising treatment for patients with metastatic gastric cancer [111]. Despite promising clinical trials results over Neo-MoDC vaccines combined with immune checkpoint inhibitors (ICT), these strategies face currently challenges, including the complex and potentially costly process of creating personalised neoantigen vaccines, scalability issues, and the potential for adverse immune reactions. Despite these hurdles, the potential for improved DC vaccines in cancer treatment is clear, with further research needed to address these challenges.

Building on these advances in DC vaccination, the response to anti-CTLA-4 blockade in poorly immunogenic tumours is contingent on DC activation, which was previously accomplished through GM-CSF vaccination, mimicking a typical ‘hot’ tumour state [20, 21]. Known as the GVAX vaccine, this approach uses genetically modified whole tumour cells to express and secrete GM-CSF. GVAX has provided consistent evidence of immune activation and clinical activity with radiologic responses across multiple cancer types in both early and late stages. Several clinical trials have been conducted using the GVAX approach to unleash immunogenicity in ICT. GVAX significantly improved the survival response of ipilimumab but not nivolumab in a cohort of pancreatic ductal adenocarcinoma (PDAC) patients, experiencing a net diversification of their peripheral TCR repertoires [62]. Notably, other clinical studies using GVAX in cold tumours, such as colon cancer and PDAC, did not only observe significant OS improvements of ICT (NCT01896869 and NCT02243371), but also evidenced significant immunological changes in the cancer TME of patients, suggesting that further studies with novel combinations in similar directions will likely support the clinical management of cold tumours. Three clinical trials using GVAX alone or combined with ICT are currently in the recruitment stage (NCT04239040; NCT03767582; NCT01952730). However, in a phase-II study comparing GVAX and ipilimumab maintenance therapy with ongoing chemotherapy in metastatic pancreatic ductal adenocarcinoma, the GVAX approach failed to improve overall survival, even indicating numerically inferior results [112]. While GVAX holds considerable promise in combination with ICT, it’s plan future enhancements to bolster its efficacy across varied cancer treatment scenarios is required. Understanding its inconsistent efficacy in improving OS across diverse cancer types can involve stratifying patients by tumour characteristics, genetic profiles, or immune status, and pursuing biomarkers predictive of GVAX responsiveness for a more targeted, personalised therapy. Personalising GVAX therapy, while crucial, presents challenges due to the genetic diversity among tumours and individuals, necessitating an investment in innovative technologies like next-generation sequencing and artificial intelligence to facilitate patient stratification and treatment customisation. By strategically addressing these issues, GVAX has the potential to become a game-changing tool in cancer immunotherapy, as primarily observed in pre-clinical studies.

**Fig. 3:**
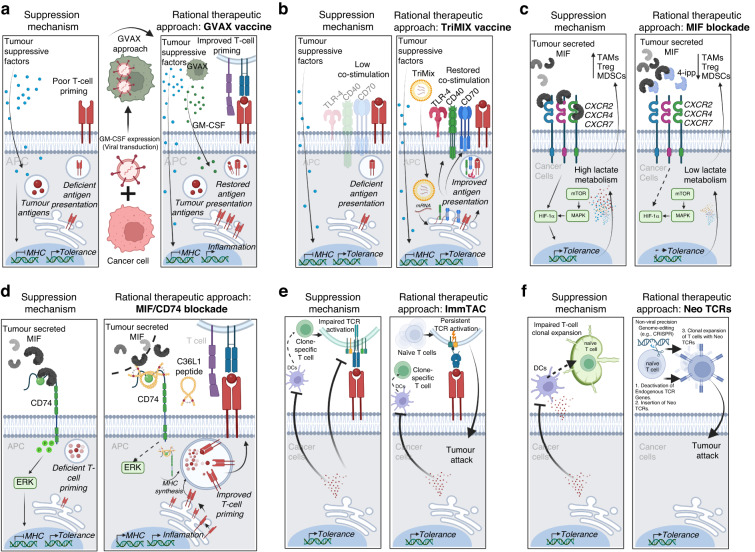
Schematic depiction of proposed novel technologies aimed at restoring T-cell generation by reinvigorating DCs immunogenicity in desert tumours. **a** GVAX vaccine enhances both systemic and localised GM-CSF levels to re-establish immunogenic functions of monocyte-derived DCs for the generation of anti-tumour T cells. **b** TriMix, an mRNA-based gene-therapy vaccine to reinvigorate the immunological synapse by restoring levels of co-stimulatory molecules in immunosuppressed DCs, thereby improving the priming of anti-tumour T cells. **c** Disrupting MIF engagement with multiple targets using 4-ipp MIF inhibitor regulates lactate metabolism within tumour cells, consequently augmenting anti-CTLA-4 response. **d** Blocking the MIF/CD74 immunosuppressive axis in DCs using C36L1 therapeutic peptide restores immunogenic phenotype and unleashes the priming of anti-tumour T cells and cancer immunosurveillance. **e** Tebentafusp triggers immune activation in T-cell desert tumours, such as mUM. **f** Clinical-grade precision genome editing facilitates the deactivation of inherent TCR genes and the integration of neoantigen-specific TCR chains for clonal expansion of anti-tumour T cells.

Adoptive cell transfer protocols using autologous DCs have also been widely investigated at the beginning of cancer immunotherapies. Today, strategies to improve adoptive DCs vaccination protocols in cancer patients include using conventional DCs, instead of monocytic differentiated DCs (which provides significant logistical challenges) [113]. CD1c+ DCs and plasmacytoid DCs previously loaded with tumour antigens adoptively transferred to Stage IV skin melanoma patients result in an improved generation of CTLs associated with improved progression-free survival (PFS) [114, 115]. To boost the maturation of DCs, which is a major factor dictating the success of DCs vaccines, DCs need to express co-stimulatory molecules, such as CD40 and CD40L. mRNA transfection-based delivery protocol for co-stimulation of CD40L, CD70 and TLR-4, has been developed to create more effective functional DCs. In combination, this mRNA vaccination strategy is called the TriMix strategy to mature DCs (TriMix-DCs) [116] (Fig. 3b). The feasibility and safety of TriMix-DCs have been confirmed, as well as their ability to elicit specific immunogenic responses [117, 118]. A recent phase-II clinical trial showed that the TriMix-DCs vaccine induced more robust immune responses in patients with complete or partial response in combination with the anti-CTLA-4 ipilimumab [119].

Since the development of anti-CTLA-4 blockade, mechanisms that would induce T-cell desertification in B16 melanoma models (albeit having significant TMB) were unknown [21]. The pre-clinical version of the GVAX-GM-CSF vaccine was the immediate solution to unleash immunogenicity for anti-CTLA-4 blockade in this melanoma model [21]. MIF has been described to suppress immunogenicity in metastatic B16 models and human UM, which are universally refractory to ICT [27, 61]. In cutaneous metastatic melanomas, the fact that MIF suppresses immunogenicity adaptive responses by targeting macrophages (MOs) and DCs through the CD74 receptor is a crucial realisation [23]. This study built a solid rationale to evaluate the impact of blocking MIF in the TME in combination with anti-PD-1 and anti-CTLA-4 ICT. Using ICT-responsive and resistant pre-clinical models, researchers found later that blocking MIF with a small-molecule 4-ipp in combination with ipilimumab improves the therapeutic response of anti-CTLA-4 [120]. Importantly, 4-ipp specifically blocks MIF interaction with different targets in the TME. Although MIF has been initially described to interact with CD74 [121], it is now known that MIF is a pleiotropic cytokine that binds to multiple immune receptors, such as CXCR7, CXCR4 and CXCR2, which have all been described to have a role in hypoxia upon binding with MIF [122, 123]. In this study, using 4-ipp in combination with ipilimumab, the authors conclude that improved anti-CTLA-4 anti-tumour effect is a result of reprogramming the metabolism of metastatic melanoma by reducing hypoxia, thus improving immunogenicity and anti-tumour efficacy [120] (Fig. 3c).

Although this study was originally grounded on the rationale that MIF/CD74 axis drives poor immunogenicity in metastatic melanoma, a significant limitation of this study is the lack of functional validation for the hypoxia-related results in MIF/CD74 dependent signalling. The interpretation is based solely on the broad blockade of MIF. Given MIF’s pleiotropic nature and interactions with other immune receptors, the observed effects may involve additional targets beyond CD74, which is not typically associated with metabolic control of the TME. Consequently, the specific therapeutic potential of solely blocking MIF/CD74 interaction in combination with ICT remains an underexplored area and requires more targeted validation studies. Nevertheless, the findings suggest that MIF inhibitors should be considered in future clinical trials as metabolic mediators of ICT responses.

Furthermore, obstructing MIF/CD74-induced signalling in the TME emerges as a promising method to boost melanoma immunogenicity, irrespective of a vaccination strategy (GVAX). Studies have shown that the therapeutic peptide C36L1 interferes with the interaction between CD74 and MIF. This interference essentially restores the ability of DCs to generate immunogenic responses by inhibiting MIF-induced tolerogenic signals through CD74. Consequently, this leads to the rejuvenation of MHC class II expression in antigen-presenting cells (APCs), an increase in the frequency of CD103+ DCs, and the promotion of clonal expansion of anti-tumour CD8 + T cells with T-cell cytotoxic capabilities (Fig. 3d) [27]. Another CDR cyclic peptide formulation (Rb9) also exhibited immunomodulatory effects on DCs by disturbing MIF/CD74 axis, boosting melanoma immunogenicity, when treating advanced lung metastasis [124].

Blocking specific mechanisms of immunogenicity suppression, such as MIF/CD74 in metastatic melanoma, as opposed to a ‘shotgun’ approach of promoting more GM-CSF expression to increase overall immune activity, could offer a more precise, efficient, and potentially less toxic therapeutic route. The utilisation of GM-CSF tumour vaccines, while successful to an extent, essentially coerces the immune system into a heightened state of immunogenicity. But this does not specifically target the underlying issue of tumour-induced immune subversion.

Regarding the potential of targeting immunosuppressive scavenger receptors in the TME, such as Clever-1, exciting pre-clinical combinatorial results opened the potential for investigating this rational in clinical settings [125]. Results from a recent clinical trial targeting Clever-1 (MATINS trial, NCT03733990) indicate a potential shift towards immune activation upon blocking a specific epitope in this target [126]. However, the study’s limited sample size, heterogeneous patient population, and heavily pre-treated participants restrict the extrapolation of these findings. The absence of clear clinical outcomes and a comprehensive understanding of molecular mechanisms, response rates, and predictors add to the uncertainty surrounding its applicability in clinical practice. Therefore, it is crucial to conduct more expansive research and clinical trials to validate Clever-1-targeting antibodies’ potential in cancer immunotherapy and address these limitations.

New therapeutic approaches are specifically needed to overcome the T-cell desertification nature of UM. A new generation of immunotherapies to overcome this deficiency is based on developing tebentafusp in cancer types with severe T-cell desertification, such as mUM [127]. This compound is a ground-breaking bispecific treatment strategy that involves a unique monoclonal T-cell receptor against cancer (ImmTAC). It targets a specific peptide-HLA complex on the tumour-cell surface, activates polyclonal T cells to release cytokines and cytolytic mediators, and is highly effective in activating the immune system against UM (Fig. 3e). Tebentafusp has shown promising efficacies in many patients in clinical trials [97–99], and the phase III IMCgp100-202 trial evaluated tebentafusp in 378 HLA-A*02:01-positive patients with untreated mUM, comparing it to pembrolizumab, ipilimumab, or dacarbazine [99]. This represents the most significant therapeutic advancement in managing mUM to date.

While the initial findings with tebentafusp are promising, its optimal utilisation still needs further research to understand better its efficacy and safety profile in a broader population of mUM patients. The current study does suggest the potential of tebentafusp to enhance survival in mUM patients; however, a more in-depth analysis is necessary to understand the underlying mechanisms. Unexplained discrepancies, such as the disconnect between survival improvements and traditional radiological indicators, indicate that we might need to look beyond conventional metrics for evaluating this treatment. Personalising the use of tebentafusp based on individual patient responses and genetic diversity within the UM population could lead to more effective therapeutic strategies. Long-term safety studies, large-scale longitudinal studies, and exploring patient-specific factors influencing the treatment response will be crucial to validate and expand on these initial findings.

Moreover, the therapeutic landscape of mUM is not confined to the potential of tebentafusp alone. Advancements in targeted therapies present additional pathways to disrupt the proliferation of UM cells and counteract their metabolic mechanisms to suppress local immune responses. For instance, darovasertib, a protein kinase C (PKC) inhibitor, has demonstrated potential in hampering UM cell growth, thereby potentially enabling immune responses to act more effectively against the disease [128]. The implications of PKC inhibition on lipid metabolic regulation [129, 130] further expand the scope of the investigation into how the metabolism of immune responses can be modulated in the context of UM, which was recently proven to be a determining factor in high-risk mUM patients [94]. The safety and efficacy of other therapies like selumetinib, evaluated in a phase Ib trial, has also indicated promise, despite some treatment-related adverse events [131]. These alternative avenues of treatment add to the growing arsenal against mUM, underscoring the continuous efforts to innovate and improve patient outcomes in this challenging disease.

In light of these critical areas requiring further innovation, significant strides have been made in personalised cell therapy. In a pioneering example, scientists have developed a clinical-grade method that not only deactivates endogenous TCR genes but also introduces two chains of a neoantigen-specific TCR (neo-TCR) into the locus encoding TCRα. Employing non-viral precision genome-editing tools, scientists have developed a clinical-grade method that deactivates endogenous TCR genes and inserts two chains of a neo-TCR into the locus encoding TCRα. In a first-in-human phase I clinical trial, 16 patients with treatment-resistant solid cancers received up to three neo-TCR transgenic cell products. Despite anticipated side effects, this therapy proved safe (Fig. 3f). The transgenic T cells could travel to tumours and were identified at higher frequencies than native TCRs, thus overcoming T-cell desertification and exclusion [132]. This research demonstrates the viability of isolating and cloning multiple TCRs that recognise mutational neoantigens, providing a powerful new immunotherapy approach for cancer patients. Although this research provides significant insights into using CRISPR-based, non-viral knockout and knock-in editing to genetically redirect T cells to mutational neoantigens in humans, improvements in sample acquisition, the efficiency of neo-TCR isolation and cloning, and optimisation of initial cell dose and neoantigen selection are required. Furthermore, advancements in addressing neo-TCR and neoantigen variability could significantly enhance the effectiveness of this personalised treatment approach.

Finally, other advances have underscored the potential of combinatorial approaches to improve ICT responses in pre-clinical and clinical settings. Integrating VEGF-targeting therapies with ICT has exhibited a noteworthy anti-tumour synergy [133, 134]. Similarly, the strategic targeting of immunosuppressive elements such as IL-10 and STAT3 has shown potential to combat immune desertification, especially when combined with ICT [40, 135]. The role of TBK1 as an immune-evasion gene has been elucidated, revealing that its inhibition can augment the response to PD-1 blockade therapy and sensitise T-cell immune effector functions [136]. Furthermore, preserving the anti-tumour activity of T cells and NK cells in MHC-I deficient tumours is achievable through a vaccine approach that targets MICA and MICB stress proteins [137]. Initiatives like the Phase I clinical trial combining bevacizumab and ipilimumab have displayed improved immune regulation and lymphocyte trafficking in metastatic melanoma [138]. Moreover, the innovative application of a bifunctional antibody, Y-trap, capable of targeting both TGFβ and CTLA-4, has also proven effective in mitigating immune tolerance and augmenting tumour-infiltrating lymphocytes [139]. These innovative approaches, alongside ICT, suggest more potent cancer combinatorial strategies, showing feasibility and promise in a clinical setting towards personalised treatments. However, these studies frequently report severe adverse events and overlapping toxicities, challenging their application. They seem to benefit only a small subset of patients, suggesting limitations in their effectiveness. Despite positive initial results, they demand further validation through long-term outcome data and broader population studies, reflecting the emerging stage of these therapies and inherent uncertainties. Therefore, despite their promise, these methods still need further confirmation of their safety and effectiveness.

## Conclusions

It is now clearly established that the ever-evolving landscape of cancer immunotherapy unveils the pivotal role of DCs dynamics in antigen processing and priming of T lymphocytes as an immunosurveillance step of supreme importance, shaping ICT efficacy within cold tumours. Accordingly, there is an increasing effort to develop combinatorial approaches to control these dynamics. The current combinatorial approaches are based on pharmacological strategies that target specific innate checkpoint regulators induced or secreted by tumour cells in cold tumours, suppressing immunogenic responses from local DCs. Furthermore, novel autologous DCs vaccination strategies and gene-therapy approach using mRNA vaccines offer the potential for enhancing T-cell priming and co-stimulation. The continuous growth of the immuno-oncology field highlights the importance of understanding the molecular mechanisms underlying T-cell desertification and exclusion, particularly related to tumours’ genetic instabilities driving cold tumours. Further research exploring the diversity of tumour’s secretomes and utilising epigenetic evidence from refractory biopsies holds the potential to uncover new insights. These findings will likely contribute to the development of next generation of innate immunotherapies aimed at unleashing immunogenicity in refractory cold tumours, complementing ICT and improving overall responses in cancer patients. With ongoing scientific advancements and a comprehensive understanding of tumour-immune interactions, the future holds promising prospects for more personalised and effective combinatorial immunotherapies.

## Data Availability

Not applicable.
